# A practical approach to imaging the axilla

**DOI:** 10.1007/s13244-014-0367-8

**Published:** 2014-12-23

**Authors:** V. Dialani, D. F. James, P. J. Slanetz

**Affiliations:** 1 Department of Radiology, Beth Israel Deaconess Medical Center, 330 Brookline Avenue, Boston, MA 02215 USA; 2 Jefferson Radiology, 85 Seymour Street, Suite 200, Hartford, CT 06106 USA

**Keywords:** Mammography, Axilla, Targeted ultrasound

## Abstract

Imaging of the axilla typically occurs when patients present with axillary symptoms or newly diagnosed breast cancer. An awareness of the axillary anatomy is essential in order to generate an accurate differential diagnosis and guide patient management. The purpose of this article is to review the indications for axillary imaging, discuss the logistics of the scanning technique and percutaneous interventions, and present the imaging findings and management of a variety of breast diseases involving the axilla.

*Teaching points*

• *Knowledge of normal axillary anatomy aids in determining the aetiology of an axillary mass.*

*• The differential diagnosis of an axillary mass is broad and can be subdivided by the location of the lesion.*

*• Imaging evaluation of the axilla usually entails diagnostic mammography and targeted ultrasound.*

*• FNA or core needle biopsies are safe and accurate methods for diagnosis and guiding management.*

## Introduction

Over the past several decades, coinciding with the introduction of ultrasound, radiologists have increasingly imaged the axilla in females presenting with axillary symptoms and newly diagnosed breast cancer. In addition, image-guided axillary procedures have been embraced as a means to diagnose findings and guide patient management. The purpose of this article is to review the indications for axillary imaging, discuss the logistics of the scanning technique and percutaneous interventions, and present the imaging findings and management of a variety of breast diseases involving the axilla.

## Basic anatomy

**Fig. 1:**
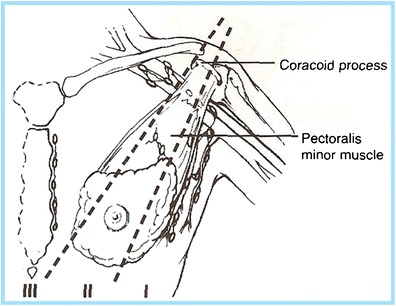
The axilla is composed of the axillary artery and vein, brachial plexus, lymph nodes, fat, accessory breast tissue, skin and subcutaneous glands. The lymph node-bearing area is divided into three regions: level I: lymph nodes lateral and inferior to the pectoralis minor muscle; level II: lymph nodes under the pectoralis minor muscle; level III: lymph nodes deep and medial to the medial border of the pectoralis minor muscle

The axilla is composed of the axillary artery and vein, brachial plexus, lymph nodes, fat, accessory breast tissue, skin and subcutaneous glands. The anatomic boundaries are as follows: superior: the clavicle, scapula and first rib; posterior: subscapularis, teres major and latissimus dorsi muscles; anterior: pectoralis major and minor muscles; medial: serratus anterior and first four ribs; lateral: coracobrachialis and short head of the biceps muscle. Generally, the breast imager is primarily concerned with the lymph nodes located in the axilla (Fig. 1). The lymph node-bearing area is divided into three regions:

Level I: Lymph nodes lateral and inferior to the pectoralis minor muscle
Level II: Lymph nodes beneath the pectoralis minor muscle
Level III: Lymph nodes deep and medial to the medial border of the pectoralis minor muscle

## Scanning technique, logistics and image-guided interventions

The standard imaging evaluation of a palpable axillary lump for females over the age of 30 years in most of the USA typically includes a mammogram with a “skin marker” overlying the palpable finding followed by a focussed ultrasound of the area of concern. This may vary in different countries and typically in the UK, for example, mammographic evaluation begins at age 40 years. In younger patients, imaging begins with ultrasound. For patients with a focal lump, scanning is typically targeted over the area of concern; however, if there is a suspicious finding, it is important to cover the entire axillary region to evaluate for any associated findings. When the study is being performed in a female newly diagnosed with breast cancer, imaging should be performed broadly to include the entire extent of the axillary contents. Examination of level I lymph nodes necessitates visualising the axillary vessels as well as scanning the entire fatty contents from the margin of the pectoralis muscles anteromedially to the latissimus dorsi and teres major margin posterolaterally. The lateral thoracic and thoracodorsal arteries and smaller branches will be seen variably along each margin, respectively. Although node groups typically follow these vessels, lymph nodes are frequently found in isolation within the axillary fat. It is also very important to scan inferiorly, down through the axillary tail, because abnormal nodes are frequently found in this location as well. Levels II and III are not routinely scanned, although large level II nodes can sometimes be seen [1].

Axillary ultrasound should be performed using a high-frequency (7.5–17-MHz) linear-array transducer. A lower frequency (5–7.5-MHz) setting may be needed for larger patients or for patients with a large axillary fat pad; however, the spatial resolution will be compromised. The patient should lie in a supine oblique position, with her hand above her head with the arm abducted and externally rotated (“bathing beauty” position). All findings should be documented in orthogonal planes with and without calipers and a lesion’s largest dimension should also be recorded. Some facilities employ grey-scale and colour Doppler US or power Doppler US during the study, especially when assessing lymph nodes. When colour Doppler US is used, it is important to use low wall filter settings and low velocity settings (higher pulse repetition frequency) to detect abnormal blood flow. The colour gain should be increased high enough to detect subtle flow without causing a colour noise artefact. When evaluating lymph nodes, ultrasound is 94 % sensitive and 72 % specific in characterising nodes as suspicious or benign based on size and morphologic features [2].

If the initial imaging evaluation of the axilla reveals a suspicious finding, percutaneous procedures, such as ultrasound-guided fine-needle aspiration (FNA) or ultrasound-guided core biopsy, may be performed. At most institutions, FNA is currently the preferred approach as the procedure is usually diagnostic and potential complications are minimal (bleeding, infection, non-diagnostic sample). FNA is typically performed using 22–25-gauge needles with aspirates sent to cytology. In most cases, at least three passes are made, especially if the cytologist is not on site during the procedure. If lymphoma is of clinical concern, additional aspirates should be obtained for flow cytometry. However, limitations do exist for FNA as it can be operator dependent, requires access to reliable cytology and carries a relatively high false-negative rate of 12–23 % [3] . Most commonly performed for suspicious lymphadenopathy, the confirmation of metastatic involvement in a lymph node precludes sentinel lymph node biopsy in females with newly diagnosed breast cancer leading to full axillary node dissection. In one study of 224 patients, the sensitivity of ultrasound-guided FNA for suspicious, indeterminate and normal-appearing axillary nodes was 93, 44 and 11 %, respectively, with an overall sensitivity of 59 % and overall specificity of 100 % [2]. The likelihood of a positive FNA correlated directly with the size of the primary breast malignancy, being 29 % for tumours <1 cm, 50 % for tumours 1–2 cm, 69 % for tumours 2–5 cm and 100 % for tumours >5 cm [2]. Alternatively, percutaneous core needle biopsy using 12–18 gauge spring-loaded or vacuum-assisted devices can safely be performed [4–7]. The risks of axillary core biopsy are similar to other image-guided procedures, namely bleeding and infection. However, given the presence of axillary vessels and nerves coursing through the axilla, a “no-throw” technique is preferred as this minimises the risk of haematoma or unintended nerve damage. If a spring-loaded device is used, this most commonly is performed on masses that exceed the throw of the device so that the needle does not pass into unaffected axillary tissue, thereby minimising the risk of unintended complications. In one study of 39 patients with suspicious lymphadenopathy, axillary core biopsy revealed 90 % sensitivity, 100 % specificity and 92 % accuracy [8] without any clinically significant complications. By recognising that the nerves course alongside the axillary vessels, the risk of bleeding and untoward nerve injury can easily be avoided in almost all cases. Although core biopsy may yield a benign aetiology, there is conflicting evidence concerning whether this approach is substantially better than FNA as the upgrade rate on surgical excision ranges from 0 to 36 % in several studies, possibly related to the small cohort size and inherent sampling error [8].

## Differential diagnosis of axillary findings

There are a wide variety of imaging findings that can be seen while scanning the axilla. Ranging from skin lesions to abnormal lymphadenopathy to posterior chest wall masses, the astute radiologist must apply their anatomic and medical knowledge to recommend the most appropriate management. Findings typically fall into six categories: skin lesions, congenital and developmental anomalies, infectious, inflammatory and metastatic lymphadenopathy, post-operative changes, benign neoplasms and extra-axillary masses (Table 1). Each of these categories is covered in greater detail below.

**Table 1:** Axillary findings

	Benign	Malignant
Skin and subcutaneous tissues	Seborrheic keratosis, sebaceous cyst, epidermoid inclusion cyst	Skin metastases, melanoma
Accessory breast tissue	Fibroadenoma, pseudoangiomatous stromal hyperplasia (PASH), fibrocystic change	Breast cancer
Lymph node enlargement	Hyperplasia/reactive node, HIV/immunocompromised state, granulomatous disease-sarcoidosis, lymphoma/leukaemia, Castleman’s disease	Primary breast malignancy, metastatic disease (breast, lung, ovarian, gastric, melanoma)
Lymph node calcifications/punctuate densities	Granulomatous disease, gold therapy (rheumatoid arthritis treatment)	Ductal carcinoma in situ (DCIS), metastatic disease [thyroid (papillary), ovarian]
Post-operative findings	Skin thickening, lymphoedema, post-operative fluid collections/lymphocele, fat necrosis, abscess, haematoma	Primary or metastatic recurrence
Neoplasms	Granular cell tumour, schwannoma	Primary breast cancer

## Skin lesion/glands

**Fig. 2:**
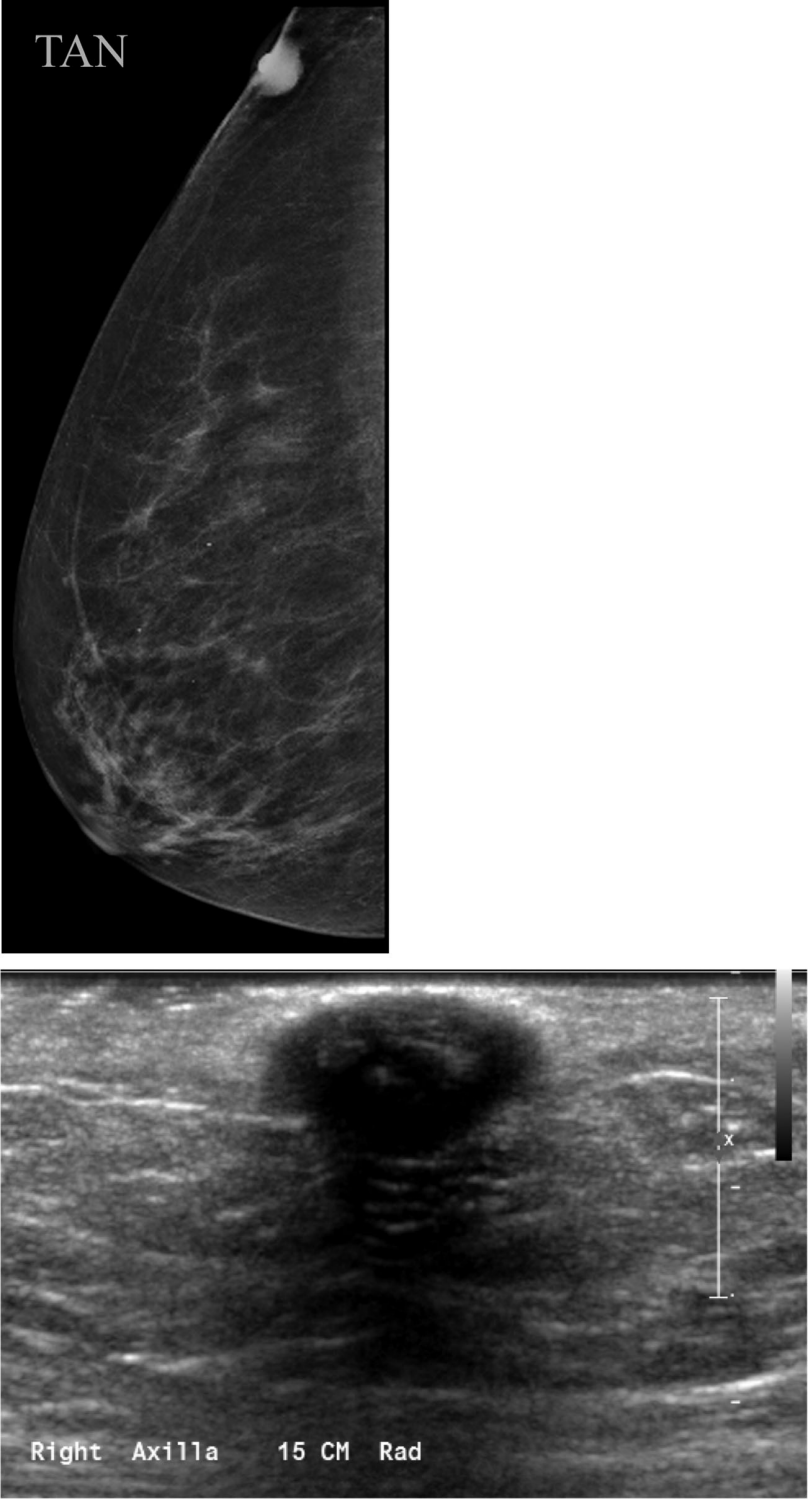
Sebaceous cyst or epidermal inclusion cyst. **a** Tangential view reveals that this lesion is located within in the skin. **b** Ultrasound confirms a hypoechoic mass within the skin

**Fig. 3:**
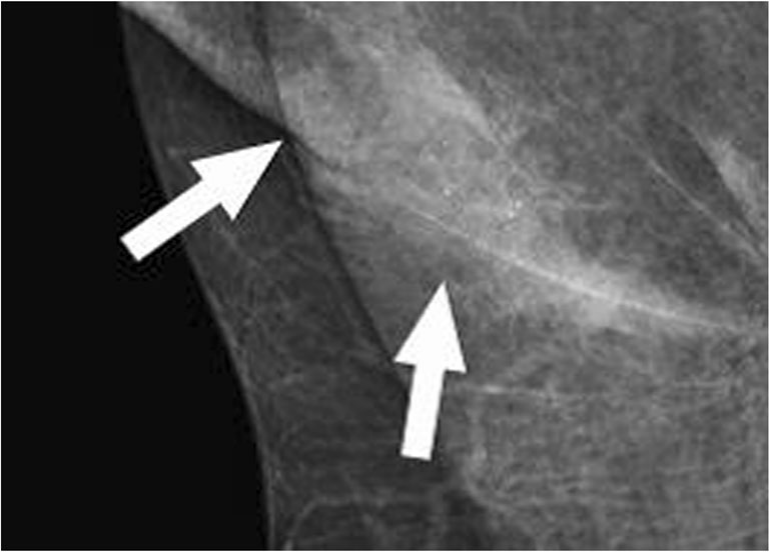
Antiperspirant artefact: magnified view shows radiopaque densities overlying the axilla

The most common skin lesion is seborrheic keratosis, a raised benign skin lesion with a verrucoid appearance. Epidermal inclusion cysts and sebaceous cysts (Fig. 2) are located at the junction of the dermis and subcutaneous fat and present on imaging as circumscribed masses often with a visible tract to the skin surface [9]. Epidermal inclusion cysts may occur anywhere but tend to be more common in the axilla and the inframammary fold. In addition, antiperspirant can appear as radiopaque densities overlying the axilla seen on mammographic imaging (Fig. 3).

## Congenital developmental anomaly-accessory breast tissue

**Fig. 4:**
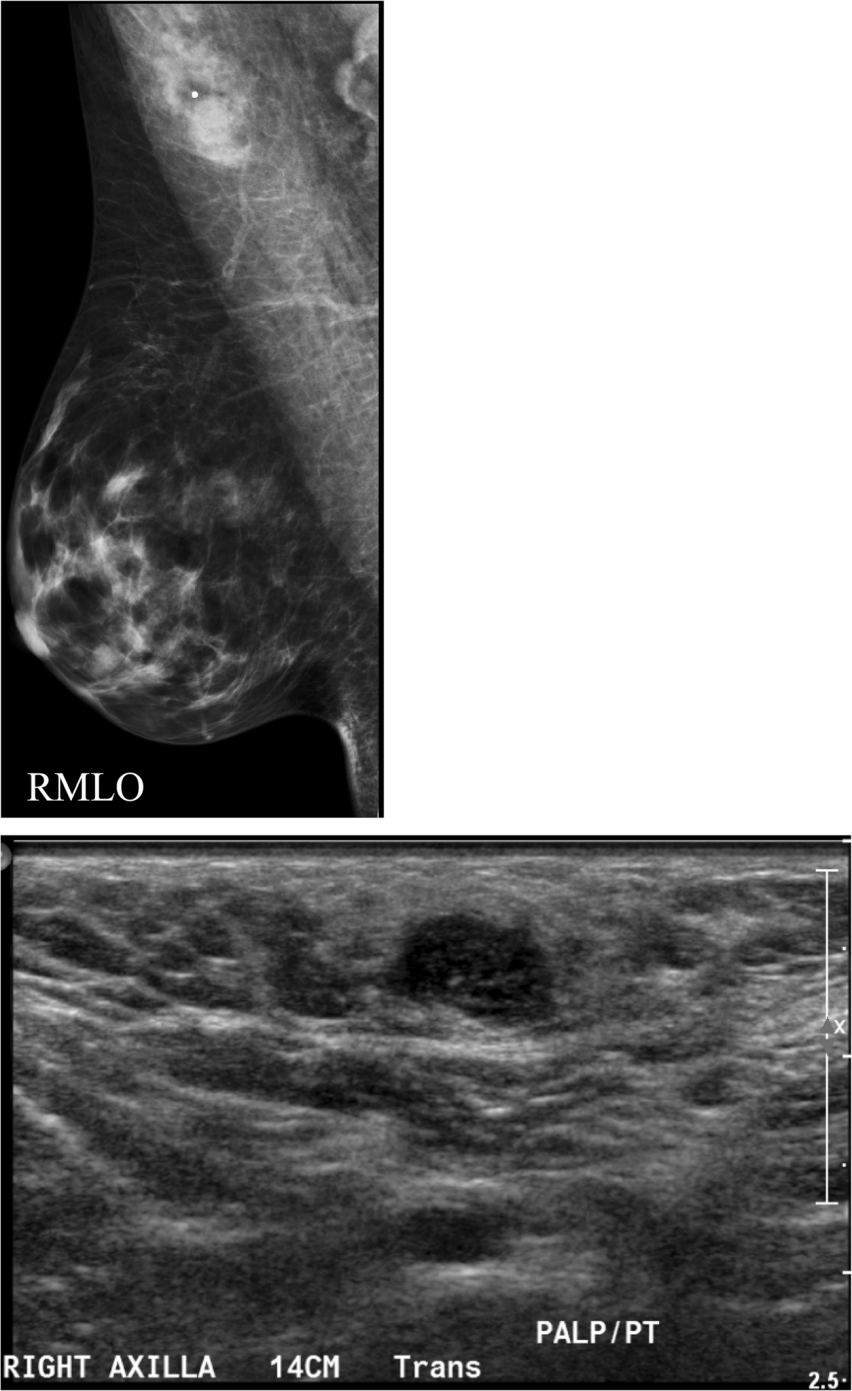
Accessory breast tissue with fibroadenoma: **a** BB placed on the skin in the area of concern to the patient. MLO views show hetereogeneously dense 4 cm asymmetry of similar density and morphology to breast tissue. **b** Targeted sonographic evaluation of the right axilla in the area of concern demonstrates fibroglandular breast tissue and a hypoechoic 8-mm solid mass. Biopsy of the nodule using a no-throw vacuum-assisted biopsy device revealed fibroadenoma

Breast tissue develops from the mammary ridges that extend from the base of the forelimb bud along the ventral surface of the embryo to an area medial to the base of the hind limb bud, which is the primitive inguinal region. Typically, the milk line regresses, leaving the middle portion of the upper third of the mammary ridge from which the breast is formed. Failure of involution of the milk line results in accessory breast tissue, most commonly in the axilla, but it can occur anywhere from the axilla to the inguinal region. Accessory breast tissue occurs in 2–6 % of women (Fig. 4) [9, 10] and can be appreciated on mammography, ultrasound and MRI. When accessory tissue occurs, both benign and malignant breast lesions can be seen (i.e. cysts, fibroadenomas, PASH, breast cancer).

## Lymph nodes

**Fig. 5:**
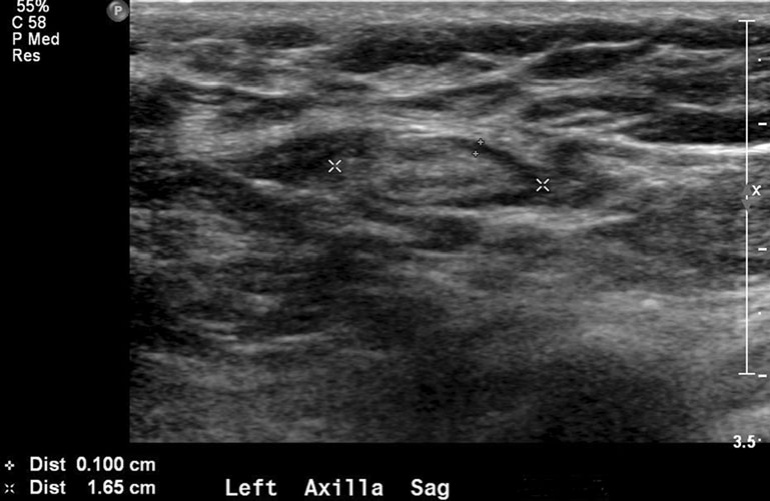
Normal lymph node: On ultrasound, lymph nodes typically are smooth, gently lobulated ovals with a hypoechoic cortex measuring less than 3 mm in thickness with a central echogenic hilum

**Fig. 6:**
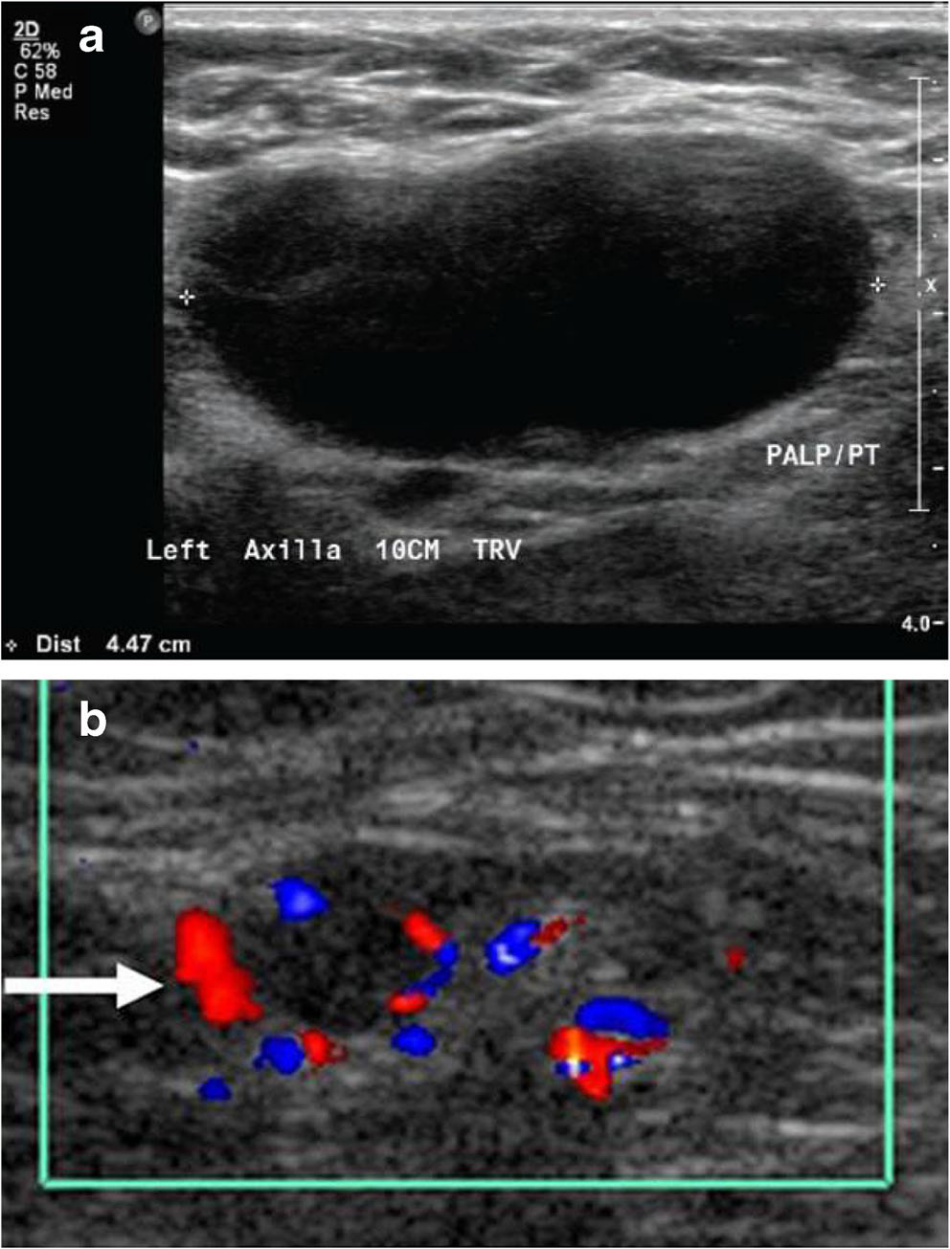
Abnormal lymph nodes: characteristics concerning for malignancy: **a** absence of the fatty hilum and **b** increased focal cortical thickness greater than 3 mm with colour Doppler US that shows hyperaemic blood flow in the hilum and central cortex or abnormal (non-hilar cortical) blood flow

Although the axilla is partially imaged on mammography, axillary lymph nodes can often be seen. Classically, benign axillary lymph nodes typically are smaller than 2 cm in maximal size and have a hilar radiolucent notch. Increased size and/or increased density of a node on mammography raises concern for pathology. However, ultrasonography provides the best means to assess the axillary nodes given its relatively sensitive and highly specific capabilities. A normal or benign-appearing axillary lymph node should have an oval or lobulated shape and a smooth, well-defined margin. The lobulated shape is because of concurrent constrictions and bulges of both the cortex and fatty hilum. The cortex should be slightly hypoechoic and uniformly thin, measuring 3 mm or less (Fig. 5). Nodes that meet this description have a very high negative predictive value for excluding metastases [11]. The echogenic hilum should constitute the majority of the node. Arterial flow in the hilum can be demonstrated with colour Doppler imaging. Morphologic criteria, such as cortical thickening, hilar effacement and non-hilar cortical blood flow, are more important than size criteria in the identification of metastases. Characteristics of lymph nodes that are concerning for malignancy besides overall size include round shape, absence of the fatty hilum, and increased concentric or focal cortical thickness greater than 3 mm, all being predictors of malignancy (Fig. 6) [12–16].

**Fig. 7:**
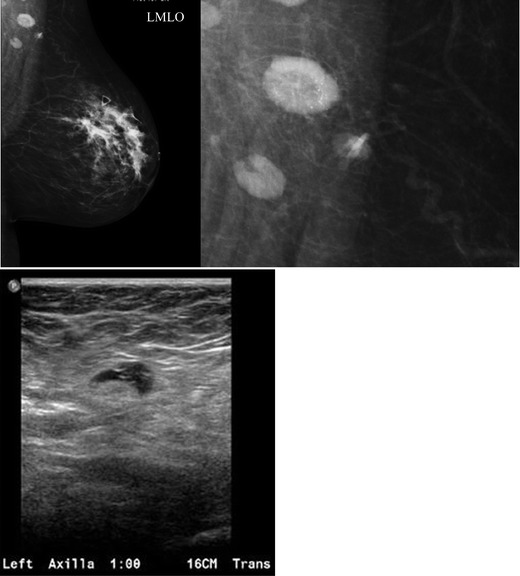
A 47-year-old female with a palpable left breast lump. **a** Left MLO view with magnified image of the left axilla demonstrates a 1.4 × 1-cm lymph node with eccentric cortical thickening and cortical calcifications. **b** Transverse image of left axillary lymph node confirms echogenic foci within the thickened cortex suspicious for malignancy. FNA was performed with aspirates sent for routine cytology and flow cytometry. Final diagnosis: metastatic carcinoma

**Fig. 8:**
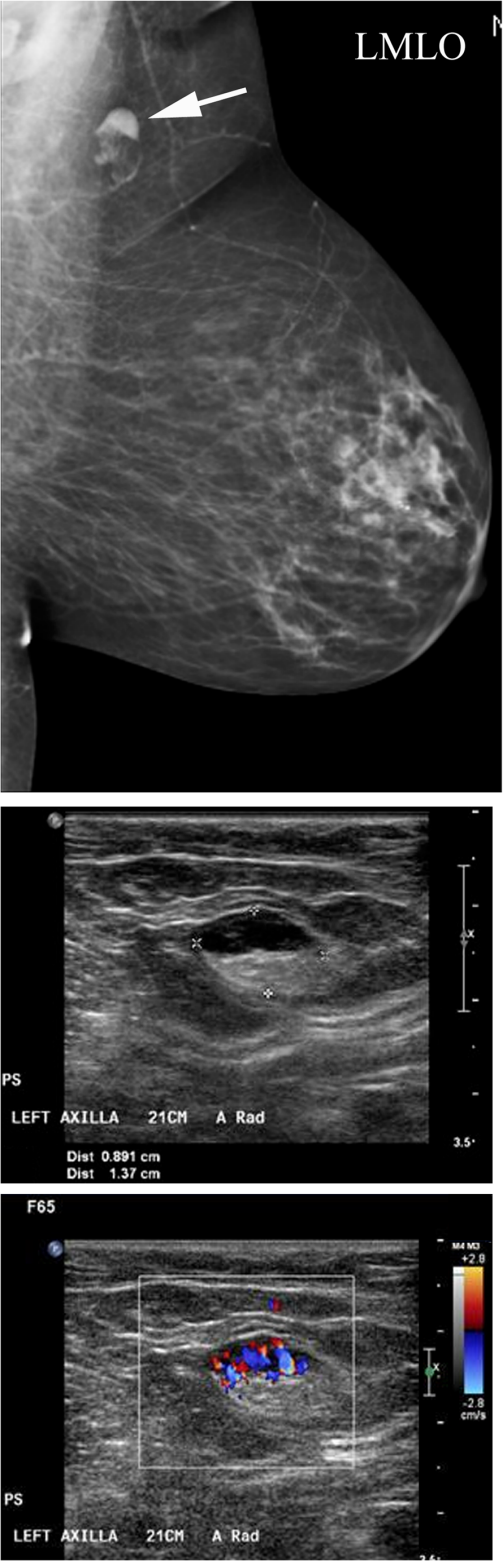
A 54-year-old female with remote history of right breast cancer and a left knee sarcoma now for screening. **a** MLO view demonstrates a focally thickened non-enlarged left axillary lymph node. **b** Ultrasound confirms a 1.4-cm lymph node with focal cortical thickening of 6 mm and (**c**) internal vascular flow. FNA was performed for diagnosis. Aspirates were sent for routine cytology and flow cytometry. Final diagnosis: reactive lymph node

A focal cortical bulge (Fig. 7) or thickening is considered the earliest detectable morphologic change in the presence of metastasis, but this criterion is difficult to apply and has a low positive predictive value because it is non-specific. This finding is therefore considered indeterminate. A true abnormal cortical bulge is seen as focal thickening of the cortex that does not follow the margin of the echogenic hilum and should be distinctly hypoechoic, and this sign is more accurate if associated with another finding such as the presence of cortical in addition to hilar blood flow. Diffuse cortical thickening can also be seen with metastasis, but this finding is even more non-specific, more often being associated with a reactive node. Eccentric cortical thickening is more suspicious than diffuse thickening, but both are non-specific and concerning enough to warrant intervention in most cases (Fig. 8). Findings seen in cases with more advanced nodal involvement, such as effacement of the fatty hilum or a rounded hypoechoic mass, have a higher positive predictive value in patients with invasive breast cancer. Replacement of the entire node or a portion of the node by an ill-defined mass is highly suspicious for malignant involvement. Occasionally, nodal microcalcifications can be seen on imaging (Fig. 7).

Contrast-enhanced ultrasonography has been used with some success to visualise enhancing abnormal nodes after periareolar injection of microbubbles. Server et al. in their study showed that sentinel lymph node can be successfully identified and biopsied using microbubble contrast-enhanced ultrasound, thereby avoiding surgical biopsy in some patients [17].

Ultrasound and axillary core biopsy enable adequate pretreatment staging in patients with breast cancer and has a positive impact on their management [18, 19]. The benefit of pre-operative identification of axillary metastases is that it allows the surgeon to proceed directly to full axillary lymph node dissection and to avoid an unnecessary sentinel lymph node biopsy, thereby avoiding a second surgical procedure involving the axillary nodes. Houssami et al. in their meta-analysis found that axillary ultrasound-guided needle biopsy (UNB) had better utility in females with average or higher underlying risk of nodal metastases [20] and found that a median proportion of 18.4 % (inter-quartile range = 13.3 %–27.4 %) from 7,097 patients can be effectively triaged to axillary treatment and avoid sentinel node biopsy (SNB). However, the changing algorithm of axillary surgical treatment means that ultrasound-guided FNA will have relatively less utility where surgeons omit axillary lymph node dissection (ALND) for minimal nodal metastatic disease. Research that allows enhanced application of ultrasound and UNB to specifically identify and biopsy sentinel nodes and to discriminate between patients with minimal versus advanced nodal metastatic involvement is likely to have the most impact on future management of the axilla in patients newly diagnosed with breast cancer [21].

Recently, the field of breast cancer surgery has seen a dramatic shift with the results of the American College of Surgeons Oncology Group Z0011 trial [22]. This study, which randomly assigned patients with sentinel lymph node (SLN)-positive breast cancer to SNB and (ALND) or to SNB alone, reported that ALND was not associated with any survival benefit and that both groups had an extremely low regional recurrence rate (0.9 % for SNB alone and 0.5 % for ALND). This was despite the fact that 27.3 % of the patients who received ALND had additional positive non-sentinel lymph nodes. This study confirmed what many surgical oncologists had suspected: that ALND provided minimal benefit while exposing a substantial number of patients to long-term morbidity, specifically lymphoedema. Therefore, there was great interest in shifting away from routine ALND for patients with SLN-positive disease, instead reserving this operation for patients who did not meet the Z0011 criteria (i.e. patients undergoing neoadjuvant chemotherapy, patients with palpable nodes, patients having mastectomy or patients with more than three involved nodes). With the results of Z0011, there has been some shift in the approach to staging the axilla. Some surgeons have advocated to no longer obtain axillary ultrasound and fine-needle aspiration (FNA) biopsy of suspicious nodes because this practice identifies women with “clinically evident” disease who, in the absence of the ultrasound, would have been “SLN positive” and thus eligible for avoidance of the ALND. However, a point to remember is that pre-operative ultrasound of the axilla is still an extremely useful staging test as nodal disease identified by ultrasound and FNA biopsy is strongly correlated with tumour burden and the number of involved nodes [23, 24].

**Fig. 9:**
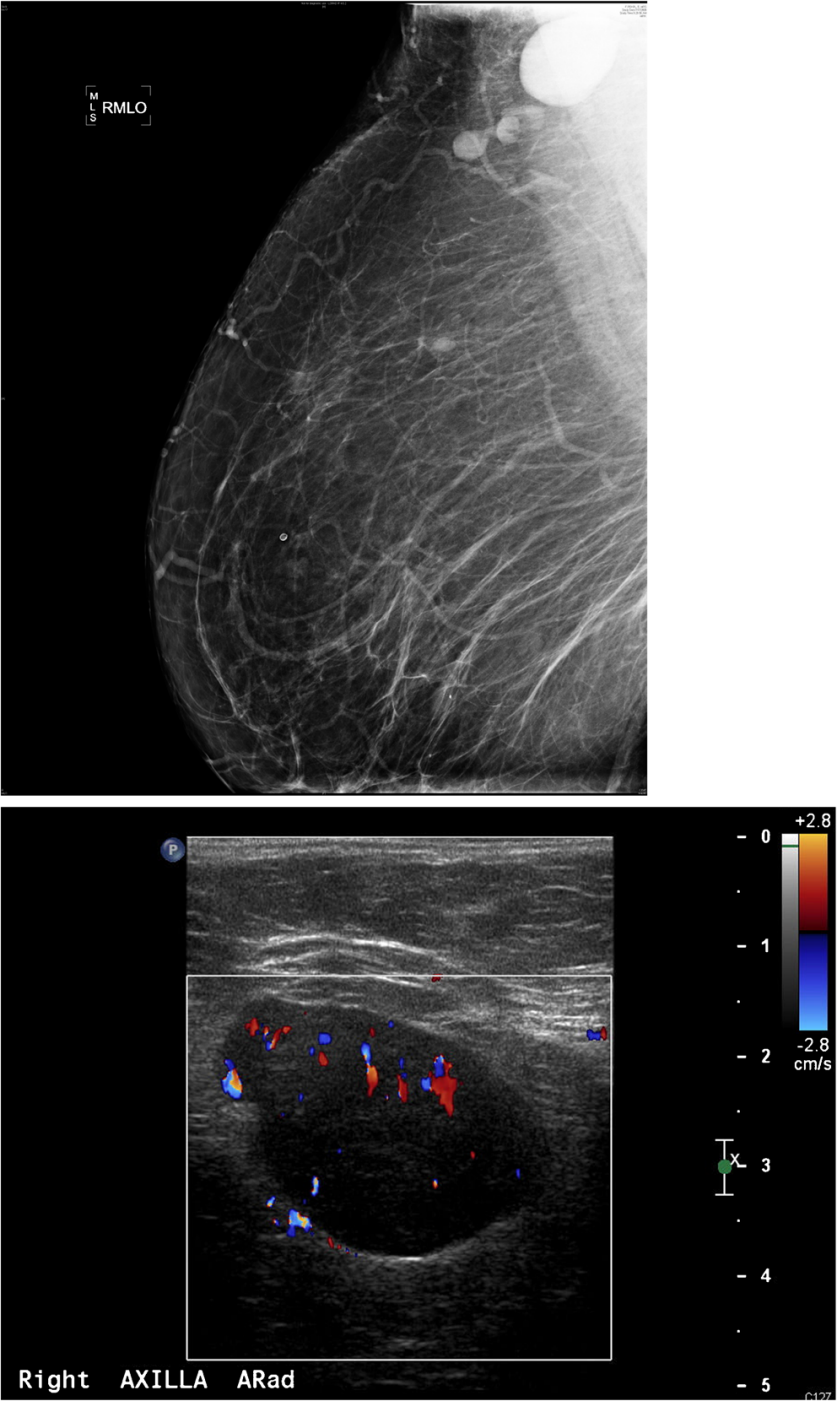
A 55-year-old African American female for screening. **a** MLO views of the breasts demonstrate a dense enlarged right axillary lymph node and several abnormal-appearing intramammary lymph nodes. Right axillary ultrasound confirms a large fatty replaced lymph node with non-hilar internal vascular flow. This is highly suspicious for malignancy in a patient with breast cancer; however this patient had a negative mammogram and so an ultrasound-guided core biopsy was performed for diagnosis. Final diagnosis: sarcoidosis

**Fig. 10:**
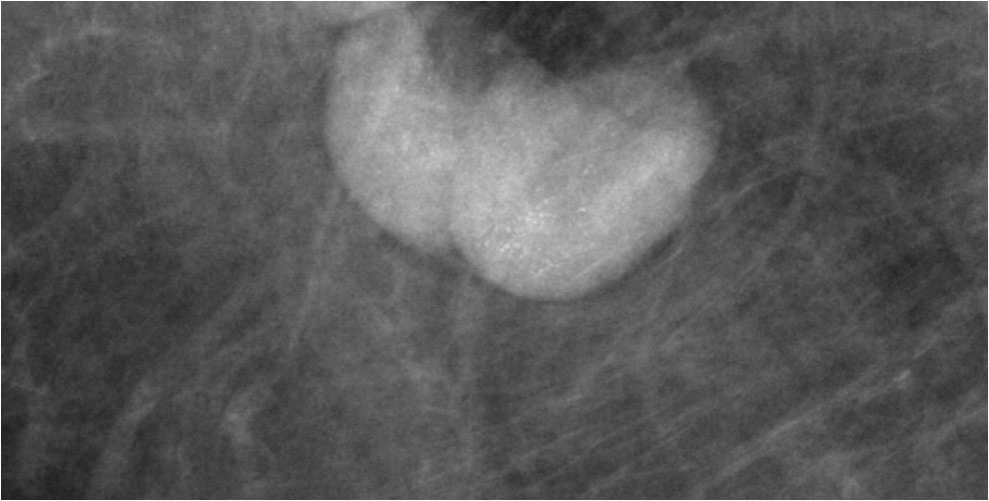
A 56-year-old female treated with gold therapy for rheumatoid arthritis. Right MLO (magnified image) of two right axillary lymph nodes reveals fine punctate radiopaque densities secondary to previous gold therapy for rheumatoid arthritis

Additionally abnormal appearing lymph nodes may be seen related to both benign (Figs. 8 and 9) and neoplastic processes (Figs. 6 and 7). In general, the differential diagnosis for an enlarged axillary lymph node includes reactive hyperplasia, HIV/immunocompromised state, sarcoidosis or other granulomatous diseases, lymphoma/leukaemia and metastatic disease (most commonly breast cancer, lung cancer, ovarian cancer, gastric cancer or melanoma). Calcifications involving lymph nodes within the axilla can be seen because of either benign or malignant causes. The differential diagnosis for calcified lymph nodes includes prior granulomatous infection, gold therapy in patients with rheumatoid arthritis (Fig. 10), collagen vascular disease, and metastasis from breast, thyroid or ovarian cancer. However, if high-density particles are seen in the axilla, the first step is to confirm that they are indeed located within a lymph node and not related to antiperspirant on the skin surface. This can readily be accomplished by recalling the patient for a repeat MLO view after cleaning the axilla to show that the densities are no longer present on the image.

## Post-operative axilla

**Fig. 11:**
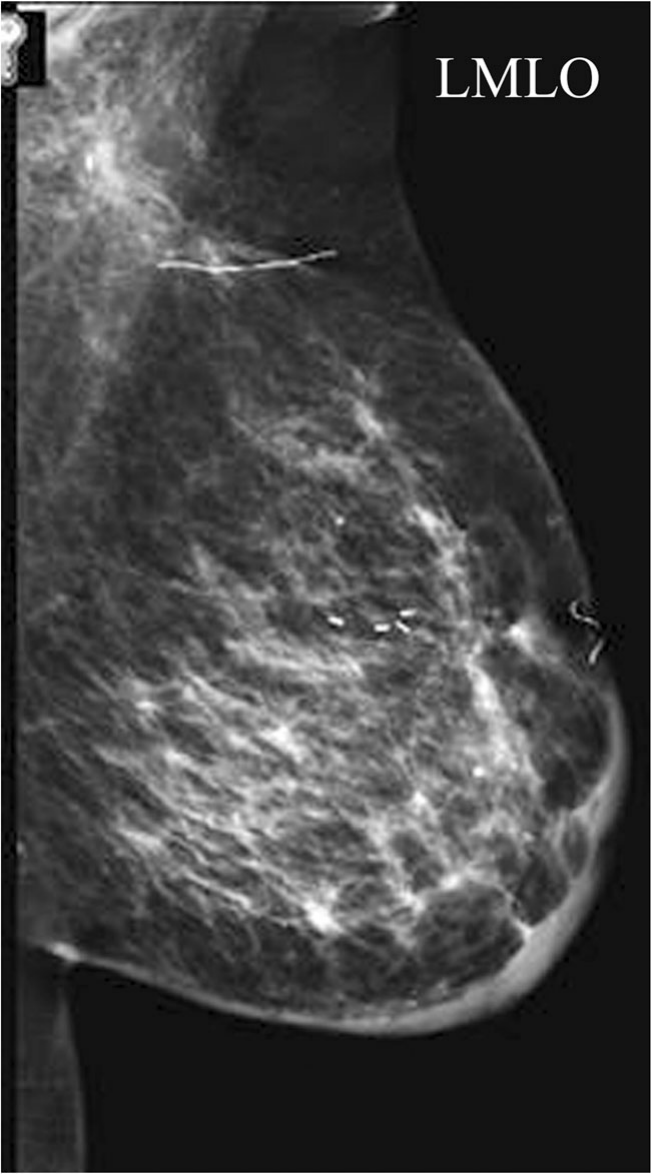
A 66-year-old female, 7 months out from lumpectomy, sentinel lymph node biopsy and radiation therapy, presents for first post-treatment mammogram. Diffuse left breast skin and trabecular thickening accompanied by post-surgical distortion in the breast and axilla are visible. Final diagnosis: Expected post-treatment changes following breast cancer diagnosis

**Fig. 12:**
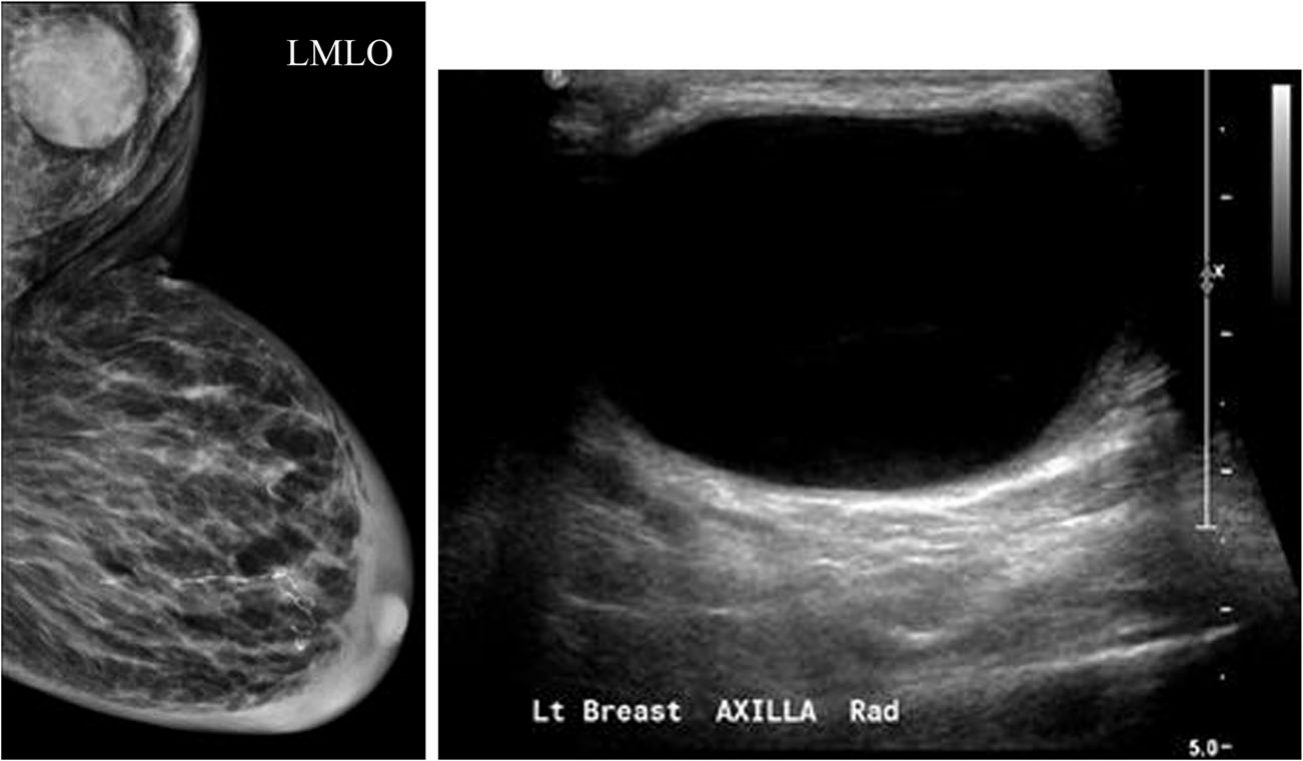
A 80-year-old female with a history of sarcoidosis presenting with a left axillary mass 1 year following axillary surgery of a benign node. **a** MLO view and (**b**) ultrasound of the left axilla demonstrates a 4.1 × 2.7 × 3.5-cm well-circumscribed predominantly anechoic structure with internal debris consistent with a complicated cyst. Aspiration was performed for symptomatic relief. Final diagnosis: lymphocele of the left breast demonstrates extensive skin and trabecular thickening and a well-circumscribed left axillary mass

Following axillary surgery, an array of findings can be seen in the axilla. The most common findings include skin and trabecular thickening (Fig. 11), post-operative fluid collections/lymphoceles (Fig. 12), fat necrosis and recurrence.

## Benign neoplasms

There are several rare benign neoplasms that can occur in the axilla, which also can mimic breast cancer in the axillary tail of Spence. These tumours are most frequently are neural in origin and asymptomatic. Therefore, they are most commonly encountered on screening mammography. As these tumours are almost always benign, percutaneous biopsy to confirm a benign entity is sufficient and most often surgery is avoided.

### Granular cell tumour (GCT)

**Fig. 13:**
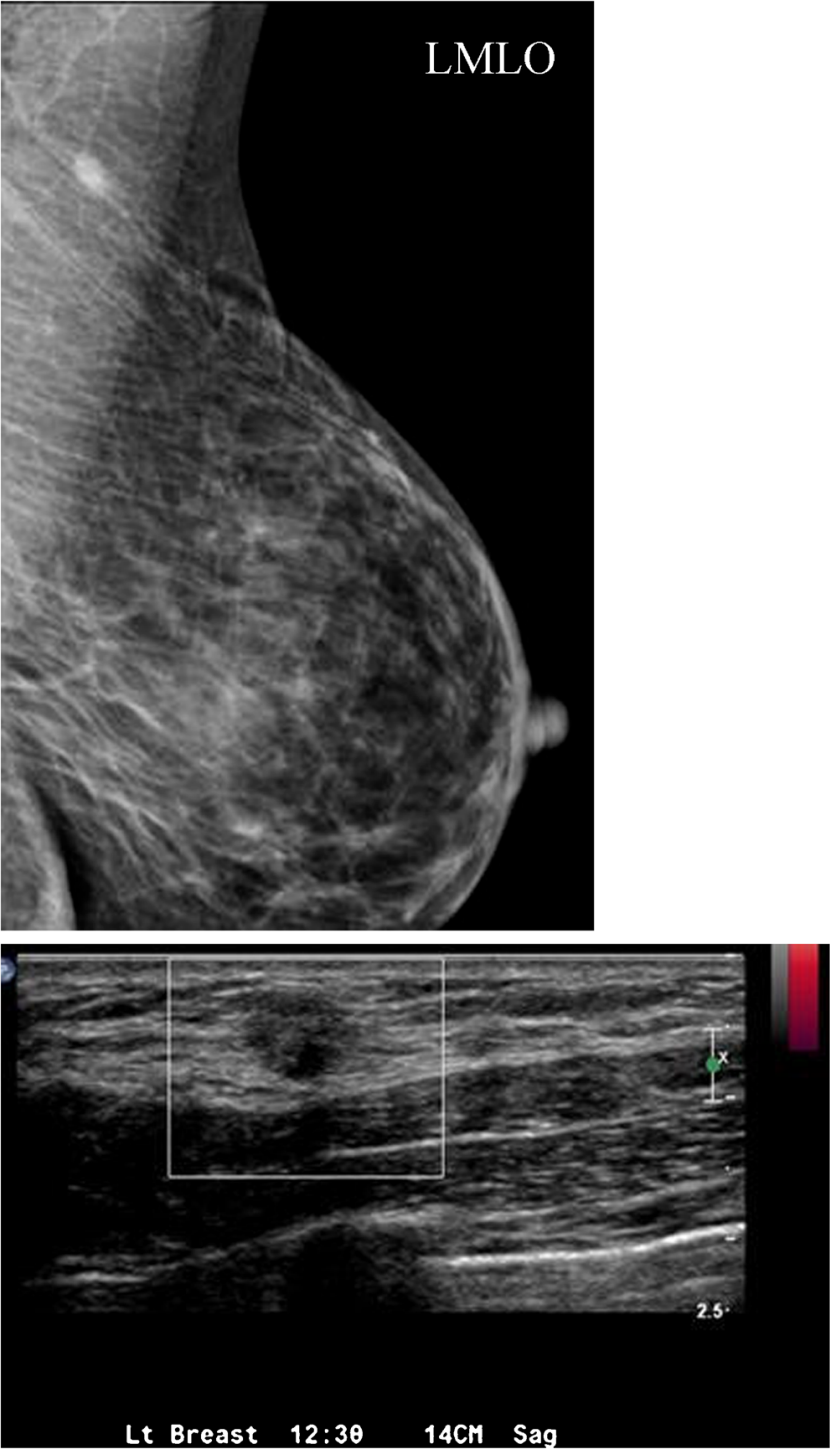
A 50-year-old female with focal 1-cm asymmetry in the left axilla region. **a** MLO views demonstrate left axillary irregular asymmetry, which persisted on spot compression. **b** Ultrasound of the left axilla demonstrated a 12-mm irregular hypoechoic hypovascular mass in the left axilla. Ultrasound-guided core needle biopsy was performed. Final diagnosis: granular cell tumour

GCT is a rare neoplasm of neural origin arising from perineural cells, accounting for less than 1 % of breast lesions and more commonly seen in African American women. Clinically, GCTs manifest as firm, palpable masses and average 1 to 2 cm in size. Histologically, they arise from the intralobular breast stroma and tend to form cords that extend into adjacent normal breast parenchyma, a characteristic that simulates the growth pattern of infiltrating carcinoma [25–27]. On mammography, granular cell tumour of the breast is difficult to distinguish from carcinoma and may have irregular or spiculated margins. On ultrasound it may be solid hypoechoic with irregular margins (Fig. 13). GCTs are usually benign neoplasms [28]. Despite the fact that most cases occur in pre-menopausal women, GCTs have been shown to be non-dependent on oestrogen and progesterone [25]. The most widely accepted theory supports a Schwann cell origin because of similarities in ultrastructural features and the positivity of the tumour for the S-100 protein, which is found in peripheral nerves. Diagnosis is confirmed by special staining for S-100 protein, NSE and CEA [28]. Less than 1 % of all GCTs, including mammary lesions, are malignant. Malignant GCTs are considered high-grade sarcomas with a high rate of metastasis and short survival. Features suggestive of malignancy include large size (>4 cm), increased mitotic rate (>2 mitosis/10 high-power fields at × 200 magnification), rapid growth, local invasion, and variation in cell size and shape. The main histological element that distinguishes GCTs from more ominous lesions is the presence of a granular cytoplasm. Current treatment is wide surgical excision. To date, there is no role for radiation or chemotherapy [26, 28]. Local recurrence has been reported after incomplete excision as late as 10 years following removal, especially when the margins are irregular or inadequate. GCTs pose a real diagnostic challenge for physicians. Pre-operative diagnosis with core needle biopsy is important because treatment is with wide surgical excision rather than mastectomy given the infiltrative pattern of growth [27].

### Schwannoma

**Fig. 14:**
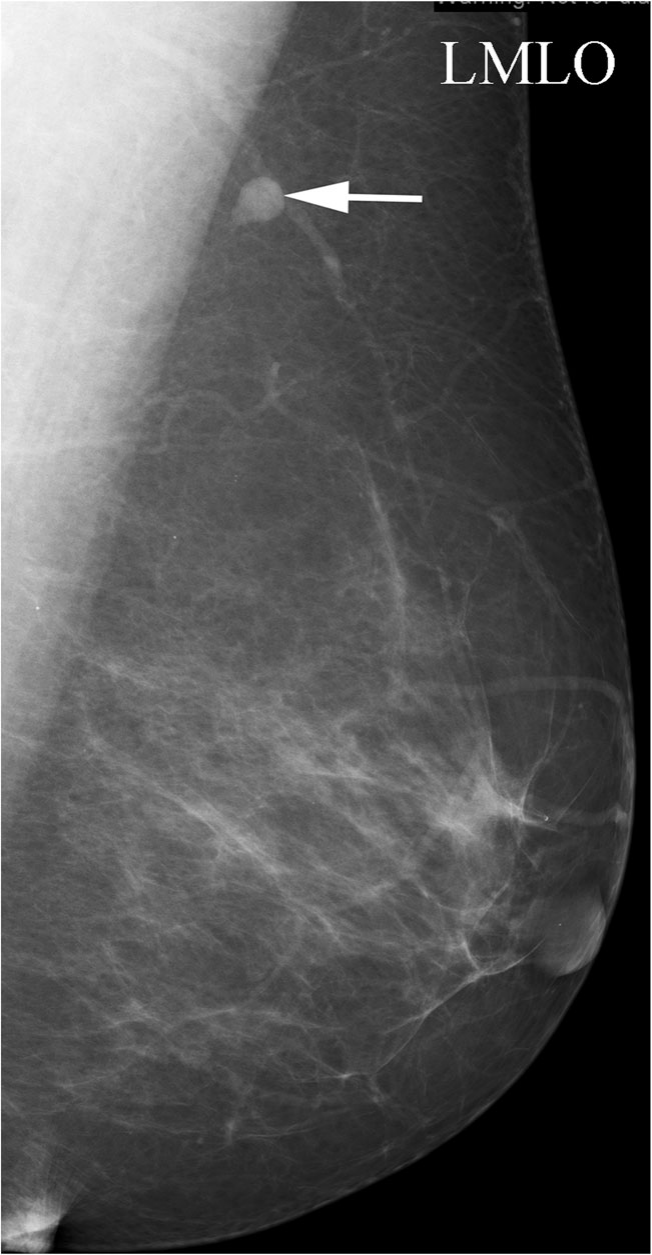
A 64-year-old asymptomatic female presented for a screening mammogram. MLO view of the left breast shows an 8-mm well-defined ovoid mass in the left upper outer quadrant (*arrow*). Final diagnosis: schwannoma

There are scattered case reports in the literature citing examples of breast schwannomas [29]. Schwannomas arise from Schwann cells of the peripheral nerve sheath. The most common locations include the head, neck and extensor surfaces of the extremities. Intramammary schwannomas account for only 2.6 % of schwannomas. Mammographically, schwannomas are most commonly described as a well-defined round or oval mass, but sometimes may be ill defined (Fig. 14). Sonographically, more variation in appearance has been reported; however, it is most commonly described as a solid hypoechoic well-defined mass with variable posterior acoustic enhancement [29]. Microscopically, classic schwannoma is an encapsulated neoplasm having two components known as Antoni A and B tissue, in variable proportions. Antoni A tissue is cellular and consists of monomorphic spindle-shaped Schwann cells, with a poorly defined eosinophilic cytoplasm and pointed basophilic nuclei, set in a variable collagenous stroma [30]. These cells commonly show nuclear palisading and parallel arrays of such palisades with intervening eosinophilic cell cytoplasm (processes) are known as Verocay bodies [31]. Breast schwannomas show no definite worrisome mammographic or ultrasonographic features, and diagnosis on imaging alone is not possible. A diagnosis of schwannoma of the breast may be suggested on core needle biopsy if there is a cytologically bland spindle cell lesion with areas of palisading and lack of epithelial elements, especially if the cells show immunostaining for S-100 protein [31]. However, distinction from other spindle cell lesions such as metaplastic carcinomas, fibroepithelial lesions with minor epithelial components, fibromatosis and myofibroblastoma, among others, is challenging and most result in surgical excision [31, 32].

## Extra-axillary lesions

**Fig. 15:**
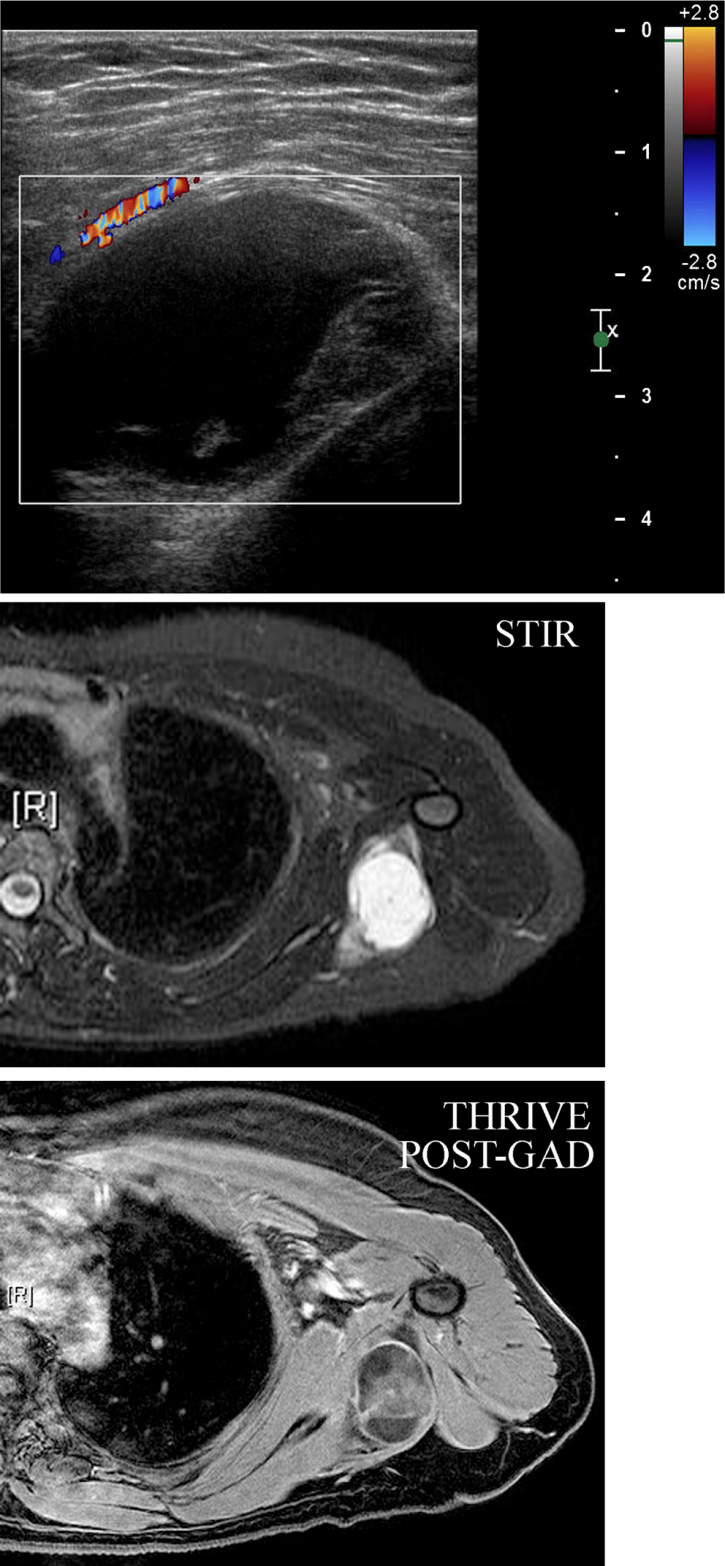
A 43-year-old female presents with a stable left axillary mass for 3–4 years, which the patient reports to fluctuate with her menstrual cycle. MLO and CC views reveal no focal mammographic abnormality (not shown here). **b** Ultrasound demonstrated a complex oval mass with cystic and solid components lying within the muscle. Colour Doppler analysis demonstrates some peripheral blood flow. **c** Axial STIR and **d** axial post-gadolinium sequences demonstrate an ovoid lesion within the latissmus dorsi muscle at the level of the axilla, which was hypointense to muscle on T1-weighted images (not shown here) and shows increased signal intensity on the fluid-sensitive STIR sequence shown here. Post-gadolinium imaging shows some internal enhancement. CT-guided needle biopsy was performed. Final diagnosis: intramuscular myxoma of the latissimus dorsi

The axilla also includes muscles and bones of the shoulder and upper rib cage. Therefore, recognising that an axillary mass is within or beneath the muscle is essential in forming an appropriate differential diagnosis. Although extra-axillary lesions are rare, benign and malignant muscular neoplasms may be encountered (Fig. 15). Intramuscular myxoma is a benign neoplasm arising from fibroblasts, which produce excessive mucopolysaccharide, accounting for their complex appearance on US and heterogeneous signal on MR imaging. Most commonly involving the heart, they can occur at other sites including subcutaneous tissue, aponeurotic tissue, bone, the genitourinary system and skin. They can be associated with fibrous dysplasia (Mazabraud’s syndrome). On imaging, intramuscular myxoma typically presents as a slowly enlarging mass that is often complex on US and most often demonstrates T1 hypointensity, T2 hyperintensity, a T1 perilesional fat rind, and rim and internal enhancement on MR. As these lesions may be indistinguishable from myxoid liposarcoma on imaging; biopsy is essential for diagnosis [33].

## Conclusions

Knowledge of the normal axillary anatomy aids in determining the underlying aetiology of an axillary mass. The differential diagnosis of an axillary mass is broad, including skin lesions, infections, haematoma, lymphadenopathy (hyperplastic, inflammatory, neoplastic or metastatic), accessory breast tissue, fibroadenoma, fibrocystic change, post-operative fluid collections, primary breast cancer and intramuscular neoplasms. For most cases, imaging evaluation entails diagnostic mammography and targeted ultrasound. If intervention is warranted, FNA or core needle biopsies are safe and accurate methods for diagnosis and for guiding the management of an axillary mass.
